# TB elimination in Southern Africa: overview and critical reflection

**DOI:** 10.5588/ijtldopen.25.0050

**Published:** 2025-07-09

**Authors:** J. Boffa, D. Vambe, C. Khosa, B. José, N. Ndjeka, T. Nkomo, A.W. Kay, A.M. Mandalakas, L. Mvusi, S.V. Omar, S. Thi, K. Velen, S. Charalambous, M.X. Rangaka

**Affiliations:** ^1^ TB Think Tank, The Aurum Institute, Johannesburg, South Africa;; ^2^ Global TB Program, Department of Pediatrics, Baylor College of Medicine, Houston USA;; ^3^ Baylor Children's Foundation Eswatini, Mbabane, Eswatini;; ^4^ Institute Nacional de Saúde, Marracuene, Mozambique;; ^5^ Department of Clinical Sciences, Liverpool School of Tropical Medicine, Liverpool, UK;; ^6^ National TB Control Programme, Maputo, Mozambique;; ^7^ TB Cluster, National Department of Health, Pretoria, South Africa;; ^8^ National TB Control Programme, Eswatini;; ^9^ Clinical Infectious Disease Group, German Center for Infection Research (DZIF), Clinical TB Unit, Research Center Borstel, Borstel, Germany;; ^10^ Centre for Tuberculosis, National and WHO Supranational TB Reference Laboratory, National Institute for Communicable Diseases a division of the National Health Laboratory Service, Johannesburg, South Africa;; ^11^ FIND, Geneva, Switzerland;; ^12^ The Aurum Institute, Johannesburg, South Africa;; ^13^ School of Public Health, University of Witwatersrand, Johannesburg, South Africa;; ^14^ Institute for Global Health and MRC Clinical Trials Unit, University College London, London, United Kingdom;; ^15^ Wellcome Centre for Infectious Diseases Research in Africa (CIDRI-Africa), Institute of Infectious Disease & Molecular Medicine and School of Public Health, University of Cape Town, Cape Town, South Africa.

**Keywords:** tuberculosis, Eswatini, Mozambique, South Africa, public health, implementation challenges

## Abstract

Despite significant progress, TB remains a major public health challenge in Southern Africa. We highlight the key initiatives in Eswatini, Mozambique and South Africa, which have implemented various interventions, including systematic TB screening, TB preventive treatment, targeted next-generation sequencing, targeted universal testing, and shorter drug-resistant and paediatric TB regimens. We also identify the key challenges, such as inconsistent drug access, increasing drug resistance and limited healthcare capacity, which continue to affect progress. Health systems must also balance TB care with broader healthcare priorities, and the integration of TB care into existing services requires further investment in outreach, treatment support and training. Identifying and treating missing people with TB, diagnosing TB in children, and improving treatment adherence remain critical areas requiring enhanced support and resources. While new diagnostic tools and treatments offer promise, their high costs and labour demands present barriers to routine implementation. Successful TB elimination will depend on simple, low-cost prevention, testing and treatment options, tailored to each country’s specific needs. All of which will require sustained political commitment, innovation and strategic investments in health system strengthening and community-based care.

Although Africa represents 16% of the world’s population,^1^ it shoulders 25% of the global TB burden^2^ and 33% of TB-related deaths.^3^ Recent advancements in testing, treatment, and prevention have accelerated the decline in TB incidence in Southern Africa, but elimination remains challenging. We examine three high-burden TB countries in Southern Africa – Eswatini, Mozambique, and South Africa – highlighting the challenges and insights gained from implementation experiences. We describe recent TB prevention and control initiatives in these countries, and identify key challenges to progress. Experts from each country were consulted, including representatives from national TB programmes, research organisations, and implementers. Through a series of meetings, these experts provided insights into national TB guidelines, strategic priorities, and examples of recent interventions, their implementation and the ongoing challenges faced. In 2023, a 5-stage TB elimination framework was proposed, recognising that a zero elimination target is not feasible due to TB reservoirs, and short-term targets will vary between, e.g., low-incidence (<10 per 100,000 per year) and high-incidence (>100 per 100,000 per year) settings.^4^ According to the WHO, the African region has an estimated TB incidence of 206 per 100,000.^5^ Eswatini, Mozambique and South Africa are all classified as WHO high-burden countries: Eswatini for TB-HIV, and Mozambique and South Africa for TB, TB-HIV and multidrug/rifampicin-resistant TB.^6^ WHO's End TB elimination targets include a 90% reduction in incidence from 2015 levels by 2035.^4,7^ Table 1 summarises the current TB burden against End TB targets for each country.^5,8^

**Table 1.** TB statistics by country.^5^

Country	Total TB incidence rate per 100 000 (2023)	End TB incidence rate target for 2035 (90% of 2015)	MD-TB incidence rate per 100 000 (2023)	HIV-pos TB incidence rate per 100 000 (2023)	HIV-neg TB mortality rate per 100 000 (2023)	HIV-pos TB mortality rate per 100 000 (2023)	TB case notifications (2023)	Treatment coverage - new & relapse (2023)	Treatment coverage - MDR/ RR-TB (2023)	Treatment success - new & relapse (2022)	Treatment success - MDR/ RR-TB (2021)
Eswatini	350 (145-507)	65	18 (7.8-29)	188 (91-318)	24 (8.9-45)	53 (25-93)	2 358	53%	33%	83%	84%
Mozambique	361 (220-537)	36	12 (6.9-18)	83 (51-124)	13 (4.7-25)	9.9 (4.9-17)	116 817	90%	32%	94%	75%
South Africa	427 (265-626)	99	21 (12-30)	230 (143-337)	39 (37-41)	49 (14-104)	222 041	79%	48%	76%	62%

MDR/RR-TB = multi-drug resistant/rifampicin resistant TB; pos = positive, neg = negative

## SYSTEM OVERVIEW AND CHALLENGES

### TB Diagnosis

In Eswatini, Mozambique, and South Africa, TB is predominantly diagnosed and treated in nurse-led decentralised public-sector clinics. Nucleic acid amplification tests (NAAT) such as Xpert MTB/Rif Ultra are widely utilised for initial testing. In South Africa, testing is being diversified with the introduction of the BD MAX™ Multi Drug Resistant TB assay (Becton Dickenson) and the Cobas MTB and MTB-RIF/INH assays (ROCHE) in addition to the placement of Xpert MTB/Rif Ultra at medium- and high-volume laboratories. Mozambique is piloting Molbio Truenat. In all three countries, Xpert MTB/XDR is replacing first- and second-line probe assays for genotypic drug-resistant TB (DR-TB) testing. DR-TB testing is decentralised, with specialised centres managing treatment and monitoring. In Eswatini, targeted next-generation sequencing (tNGS) is employed routinely when DR-TB is initially detected, with similar plans in South Africa and Mozambique. Presently, TB screening is primarily passive, relying on symptom-based assessments in clinics. Close contacts of people with TB are encouraged to undergo screening. Community-based contact tracing is a high priority in all three countries, but coverage is limited due to resource constraints. External technical agencies often bolster community-based government initiatives.

### Gaps in case estimates and notifications

TB notifications across the region align with WHO reporting standards. Eswatini reports 53% of estimated TB cases, Mozambique 96%, and South Africa 79%.^5^ Experts suggest that Eswatini’s reporting gaps arise from reliance on symptom-based screening, a high TB-HIV co-infection rate and delays in implementing strategies to detect asymptomatic TB.^9^ In Mozambique, WHO baseline TB estimates decreased between 2019–2020 following validation of a national TB prevalence survey.^10^ This combined with various interventions, such as community case finding, contact tracing, intensified TB tracking in health facilities, and the introduction of molecular tests, contributed to markedly increased notifications from 2020 (personal communication with co-author B. José). South Africa’s 2018 TB prevalence survey reported that 58.2% of people diagnosed with TB did not report symptoms.^11^ The country has seen a recent uptick in notifications, likely aided by a targeted universal TB testing strategy, described below. Nevertheless, data privacy laws in South Africa complicate programme monitoring by restricting access to individual-level data. Private-sector notifications remain challenging in all three countries, with potential for underreporting by private general practitioners, but also double counting when clients move between public and private sectors.^12^

## RECENT INITIATIVES

### TB preventive treatment

TB preventive treatment (TPT) options in Eswatini, Mozambique and South Africa include daily isoniazid (6H – also 12H in South Africa), daily rifampicin-isoniazid (3RH), and weekly isoniazid-rifapentine (3HP). These regimens are prioritised for people living with HIV (PLHIV) and household contacts, tailored by age and antiretroviral therapy.^13^ The increasing availability of 3HP along with dedicated campaigns targeting PLHIV have helped to improve TPT coverage significantly in this population. The IMPAACT4TB initiative (2018–2024) expanded 3HP access across eight sub-Saharan African countries, including Mozambique and South Africa, and contributed to a substantial price decrease from $77 to $10.^14^ Eswatini’s 100-day campaign increased TPT coverage among PLHIV from 65% in 2019 to 81% in 2023. Despite progress, challenges persist. These include healthcare worker hesitance,^15,16^ pill burden and increased clinic visits,^15,17^ although shorter regimens, improved TB-HIV integration, and options for multi-month dosing have made client access easier.^18,19^ Experts agree that more work is needed to improve TPT demand. Other barriers include the availability of rifapentine, its limited shelf-life (two years), and access to simpler fixed-dose 3HP combinations.^20^ TPT uptake among household contacts also remains low at 15% in South Africa, 17% in Eswatini, and 56% in Mozambique in 2023.^5^ Challenges include reaching contacts for screening and low demand among those offered TPT, although models for implementing community-based screening and treatment have shown promise.^21^ Access to data on TPT completion, regimen type, and adverse events is currently limited, as are data on access in other key populations, such as people with silicosis, healthcare workers, people in correctional facilities and miners.

### Screening

Digital chest X-ray (dCXR) with computer-aided detection is being introduced to detect asymptomatic and clinical TB earlier. Its appeal is the potential to screen large numbers of people at a lower cost than testing all symptomatic and high-risk people.^22^ Real-time results reduce unnecessary follow-up for people who screen negative. However, in practice, dCXR implementation has been challenging and the high initial costs for machines have created a barrier for broader implementation. Although early evidence from South Africa suggests dCXR screening can reduce testing expenditure, the volume of screens needed remains high and throughput is lower than vendor estimates.^23^ Standardising screening algorithms has also been a challenge in South Africa due to its introduction by numerous implementing agencies. The growing number of software options for interpreting results has contributed to standardisation and operational challenges^24^ making assessment of optimal dCXR placement difficult. In Eswatini, where machines are operated by the Ministry of Health, experts report issues with machine maintenance. The introduction of ultra-portable machines may address portability concerns, but regulatory obstacles remain, especially in South Africa.

### Diagnosis

**Figure 1.**
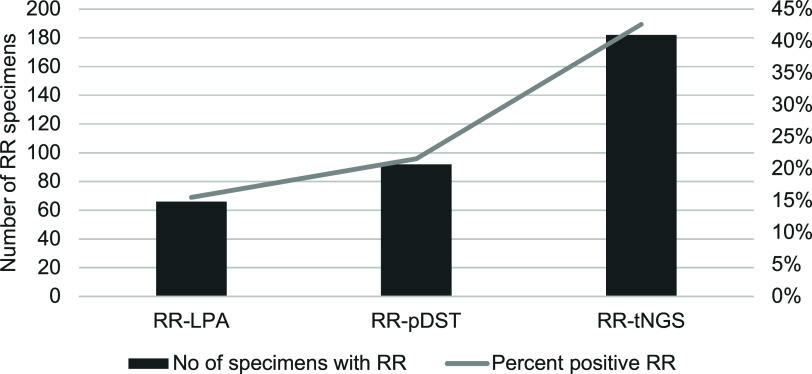
Rifampicin susceptibility by test type in Eswatini, September 2021–August 2024. RR = rifampicin resistance; LPA = line probe assay; pDST = phenotypic drug-susceptiblity testing; tNGS = targeted next-generation sequencing; No = number

The recent WHO recommendation for use of targeted next generation sequencing (tNGS) for TB care^25^ has the potential to improve diagnosis and treatment of DR-TB; however, its use is also not without challenges. In 2021, Eswatini introduced tNGS to address the emerging threat of the *rpoB* I491F TB mutation strain that cannot be detected by traditional diagnostic techniques.^26,27^ Over three years, tNGS detected rifampicin resistance in nearly twice as many DR-TB specimens (n=182/427) than Xpert MTB/Rif Ultra, line probe assay, or phenotypic drug susceptibility testing (n=92/427), see Figure 1.^28^ This led to significant treatment changes, contributing to a treatment success rate of 84% among people with DR-TB. However, 82% of *rpoB* I491F strains were associated with potential resistance to bedaquiline,^28^ raising local concerns about the introduction of shorter TB regimens. In South Africa, client specimens are sent to the National TB Reference Laboratory for Sanger sequencing to detect mutation outside the rifampicin-resistance determining region such as *rpoB* I491F in instances of first-line treatment failure or isoniazid monoresistance. Since 2017, these mutations have been observed to a much lesser extent than reported in Eswatini (personal communication with SV Omar, South African National TB Reference Laboratory). South Africa and Mozambique have both begun to incorporate tNGS in a phased approach; however, due to the placement of the technology, the *rpoB* I491F mutation will still be missed due to diagnostic escape. At this time, it is not feasible for tNGS to be applied to all presumptive TB diagnostic investigations due its high complexity and associated costs. A rapid molecular diagnostic should be developed for this purpose.

### Targeted universal TB testing

**Figure 2.**
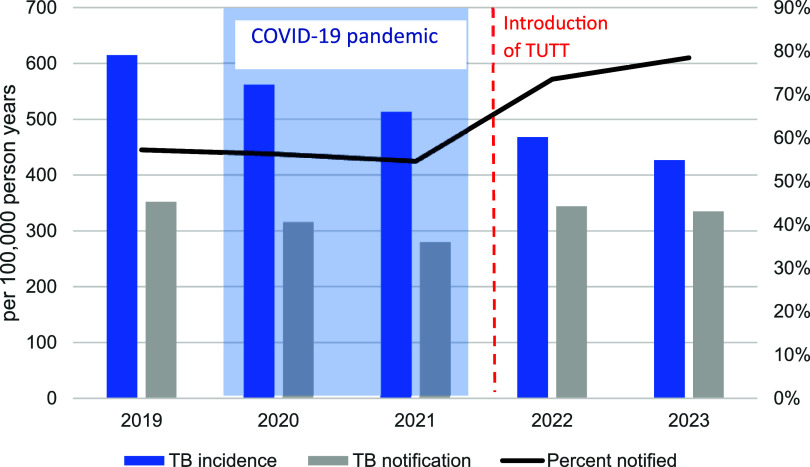
TB notifications vs incidence in South Africa, 2019–2023 (based on WHO data).

In 2022, South Africa implemented targeted universal TB testing by TB-NAAT for PLHIV, close contacts, and people with prior TB, regardless of symptoms.^29^ Annual TB testing among PLHIV has been highly effective, driven in part by routine healthcare visits for HIV treatment. Reaching contacts and people with a history of TB has been more challenging, requiring improved investment in community-based tracing. The changes to South African TB notifications between 2019–2023 is shown in Figure 2, with many experts attributing recent success to targeted universal testing.

### Treatment

Eswatini, Mozambique and South Africa have all recently introduced shorter six-month pretomanid-containing DR-TB regimens, with moxifloxacin or levofloxacin added for fluoroquinolone-susceptible clients.^30,31^ Implementation has been largely successful, but drug resistance remains a concern. A study in Mozambique reported that 61/704 rifampicin-resistant specimens were also bedaquiline-resistant on whole genome sequencing, with the prevalence increasing from 3% in 2016 to 14% in 2021.^32^ In South Africa, phenotypic results take on average 56 days to detect resistance to linezolid or bedaquiline, often delaying treatment adjustments until clients are halfway through shortened regimens. Additionally, only 47% of clients are tested for bedaquiline resistance in South Africa, primarily in high-burden areas, which may skew detection rates. Expanding tNGS testing to all drug-resistant cases could address these issues. Concerns have also been raised by experts in Mozambique about decentralised DR-TB treatment initiation and monitoring as increased regimen options and complexity require higher levels of clinical proficiency and oversight. Although decentralisation has clear benefits and remains the goal, more evidence is needed to evaluate nurse-initiated shortened DR-TB regimens.

Several new drug-susceptible TB regimens have been recently recommended by the WHO.^33–35^ A four-month paediatric regimen for non-severe TB will soon be introduced in all three countries;^36^ however, certain limitations have prevented the uptake of four-month adult regimens. A lack of available data on PLHIV,^37^ the expense of standardising fluoroquinolone susceptibility testing and greater pill burden, were all seen as limiting factors. Policymakers will instead await expanded research on the new regimens and/or the introduction of other shortened regimens that are better tailored to population demographics and testing resources.

## ADDITIONAL GAPS AND CHALLENGES

### Finding missing people with TB

**Figure 3.**
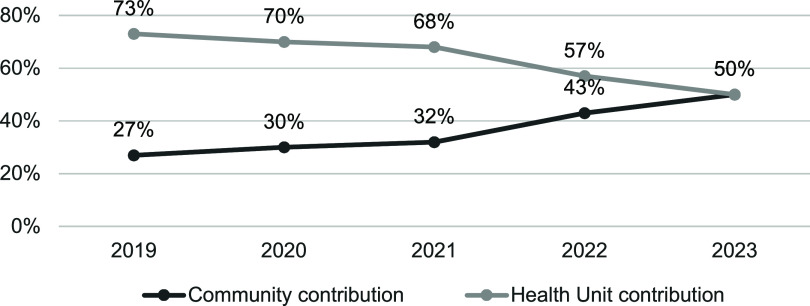
Community contribution to TB case referral and notification in Mozambique, 2019–2023.

Declining TB incidence complicates case finding. Expanding antiretroviral therapy coverage has improved detection in PLHIV, but other high-risk groups (such as children, men and the extremely poor) require targeted interventions. Community-based outreach, including community screening, house-to-house contact tracing, and follow-ups for individuals whose treatment has been disrupted, has been effective,^38^ (Figure 3) but faces resource constraints to reach scale.

### Diagnosing TB in children

Child TB notifications remain below targets in Eswatini (8% vs 12%) and South Africa (7% vs 10%), whereas in Mozambique, notifications in children are higher at 12%.^5^ Challenges arise in that children often have non-specific symptoms and difficulty providing sputum specimens.^36^ Invasive collection methods (such as sputum induction and gastric lavage) are underused due to healthcare worker discomfort. Nurses also lack confidence in making clinical diagnoses in the absence of microbiological confirmation. Stool-based diagnostics show promise,^39^ but are not yet widely implemented in Eswatini and South Africa. Promising evidence from Eswatini has shown that tNGS of stool can identify mutations predicting drug-resistance, which may be of particular benefit to children who rarely have positive TB cultures necessary for traditional phenotypic drug susceptibility testing.^40^ WHO diagnostic algorithms for children may help improve diagnosis, but require validation in primary care settings.

### Adherence support

Given the introduction of shortened regimens and rising drug resistance, adherence support is critical. Several adherence models exist, but funding to support and sustainably scale-up initiatives is lacking. In Mozambique, community self-help groups have been piloted,^38^ whereas in Eswatini, food parcels, treatment supporters, and transport reimbursement for clients with DR-TB, have been implemented. Social incentives are also being introduced in Mozambique for children under five years with TB and people with DR-TB. In South Africa, a pilot health hotline to assist clients with treatment disruptions showed promise, but lacked sustained funding. Psychosocial assessments at diagnosis are being explored to prioritise high-risk individuals. Video-observed treatment, medical monitors, behavioural nudges, and nutrition support are also under study as potential methods to improve adherence.

## CRITICAL REFLECTIONS

South Africa, Eswatini and Mozambique have made significant progress in TB prevention and care (Table 2 provides a summary of the challenges and potential solutions). Initiatives include systematic screening, TPT scale-up, tNGS, targeted universal testing, and shorter regimens for DR-TB and paediatric TB. In spite of these efforts, challenges like drug access, drug resistance, and limited human resources persist. TB incidence is declining in both people living with and without HIV, but more efforts are needed to actively find and treat TB in communities, particularly in children and vulnerable groups. The 5-stage framework for TB elimination stresses setting country-level goals that reflect each nation's context,^4^ and recommends that high-burden settings aim for a 50% reduction in incidence over five years, with priorities like improving access to care, reducing transmission, and ensuring reliable diagnosis and treatment.^4^ These actions are crucial in Eswatini, Mozambique and South Africa where gaps in care and access to services remain. Key actions from the 5-stage framework include scaling up molecular diagnosis, promoting universal TB services, and reducing losses to follow-up.^4^ These are aligned with the ongoing challenges in these countries, particularly regarding differentiated care models and TB contact tracing, which suffer from staffing shortages. Research into TB self-testing may help overcome access barriers.^41^

**Table 2.** Summary of key challenges and potential solutions.

Key aspect	Implementation challenge	Possible solutions
TB diagnosis	- Challenges with testing coverage and accuracy in some areas - Diagnostic escape (e.g. rpoB I491F mutation detection)	- Increase access to molecular tests like tNGS - Improve diagnostic infrastructure
Gaps in case estimates and notifications	- Inconsistent TB case reporting - Delays in detecting asymptomatic TB - Under reporting from private sector	- Improve case detection strategies - Increase active case-finding - Incorporate dCXR in targeted settings - Address data privacy issues for better monitoring
TB preventive treatment (TPT)	- Hesitancy from healthcare workers - Pill burden - Low uptake among household contacts	- Shorter regimens (e.g. 3HP) - Community-based screening and TPT initiation - Simplified dosing options
Digital chest X-ray screening	- High initial cost of machines/machine maintenance - Low throughput - Standardisation issues	- Address cost and maintenance challenges - Implement ultra-portable machines - Standardise screening algorithms - Increase support for threshold setting
Targeted next generation sequencing (tNGS)	- High cost of tNGS - Limited capacity in interpretation of results with clinical significance	- Develop rapid molecular diagnostics - Expand tNGS use for all DR-TB diagnoses - Build capacity to interpret sequencing results
Targeted universal TB testing	- Difficulty in reaching contacts and people with prior TB - Resource constraints for community tracing	- Increase investment in community-based tracing - Improve outreach and follow-up strategies
Shorter drug-resistant TB regimens	- Rising drug resistance - Delayed resistance testing results - Complexity of decentralised treatment	- Expand tNGS testing - Improve monitoring systems for drug resistance - Invest in diagnostic tools in parallel with new drug candidates - Enhance training and expand nurse-led decentralised treatment initiation
Shorter drug-susceptible TB regimens	- Lack of data on PLHIV - Expense of fluoroquinolone testing - Client pill burden	- Wait for expanded research on regimens - Focus on shortened regimens that match local demographic needs
Finding missing people with TB	- Declining incidence makes case-finding increasingly challenging - Resource constraints for community outreach	- Expand community-based outreach - Improve house-to-house contact tracing - Use follow-ups to reach missing people with TB
Diagnosing TB in children	- Non-specific symptoms - Difficulty in specimen collection - Underuse of invasive methods	- Implement stool-based diagnostics - Improve training for healthcare workers - Validate WHO diagnostic algorithms for children
Adherence support	- Lack of sustainable funding for adherence programmes - Challenges with client motivation	- Introduce community self-help groups - Offer tailored adherence support models - Pilot health hotlines and psychosocial assessments - Encourage shared decision-making with clients

### Increasing drug resistance and complex treatment guidelines

As the complexity of drug resistance grows, improved diagnostics are essential. The use of tNGS holds promise, but it is costly and limited in coverage. As shorter treatment regimens become available, reliable resistance profiles are necessary. Investment in diagnostic tools in parallel with new drug candidates and training of laboratory staff and clinicians is crucial for safe and effective treatment.

The frequent updates to WHO guidelines and new interventions create challenges for frontline workers. Although current interventions are beneficial, they are imperfect and costly to implement within health systems that must balance TB care with broader healthcare priorities. Countries should set their own agenda tailored to their specific needs and new tools should be prioritised based on their impact and the resources available. Increased investment is needed in health systems for basic TB care, outreach, and treatment support, as well as in training, implementation, and monitoring of new tools. Better TB vaccines are on the horizon and WHO estimates that a 50% effective vaccine could prevent 76 million TB cases and 8.5 million deaths over 25 years.^42^ Policies for equitable vaccine access and building community trust will be essential.

## CONCLUSION

Despite declining incidence, evidence from Eswatini, Mozambique, and South Africa demonstrates that meeting the End TB targets remains a challenge. Success will depend on simple, low-cost prevention, testing and treatment options – with TB self-testing and same-day testing and treatment initiation. Effective decentralisation is also vital. Adopting the 5-stage framework^4^ and focusing on realistic, country-specific goals will be key to overcoming these challenges and moving towards TB elimination.
